# Inhibitory Projections from the Inferior Colliculus to the Medial Geniculate body Originate from Four Subtypes of GABAergic Cells

**DOI:** 10.1523/ENEURO.0406-18.2018

**Published:** 2018-11-14

**Authors:** N. L. Beebe, J. G. Mellott, B. R. Schofield

**Affiliations:** Northeast Ohio Medical University, Rootstown, Ohio 44272

**Keywords:** GABA, perineuronal net, soma size, tectothalamic, thalamus, VGLUT2

## Abstract

GABAergic cells constitute 20–40% of the cells that project from the inferior colliculus [(IC) a midbrain auditory hub] to the medial geniculate body [(MG) the main auditory nucleus of the thalamus]. Four subtypes of GABAergic IC cells have been identified based on their association with perineuronal nets (PNs) and dense rings of axosomatic terminals expressing vesicular glutamate transporter 2 (VGLUT2 rings). These subtypes differ in their soma size and distribution within the IC. Based on previous work emphasizing large GABAergic cells as the origin of GABAergic IC–MG projections, we hypothesized that GABAergic IC cells surrounded by PNs and VGLUT2 rings, which tend to have larger somas, were more likely to project to the MG than smaller cells lacking these extracellular markers. Here, we injected retrograde tract tracers into the MG of guinea pigs of either sex and analyzed retrogradely labeled GABAergic cells in the ipsilateral IC for soma size and association with PNs and/or VGLUT2 rings. We found a range of GABAergic soma sizes present within the IC–MG pathway, which were reflective of the full range of GABAergic soma sizes present within the IC. Further, we found that all four subtypes of GABAergic IC cells participate in the IC–MG pathway, and that GABAergic cells lacking PNs and VGLUT2 rings were more prevalent within the pathway than would be expected based on their overall prevalence in the IC. These results may provide an anatomical substrate for the multiple roles of inhibition in the IC–MG pathway, which have emerged in electrophysiological studies.

## Significance Statement

GABAergic cells constitute a substantial proportion (20–40%) of the cells that project from the inferior colliculus to the auditory thalamus. Previous studies highlight a single subtype of GABAergic colliculothalamic cell characterized by a large soma and dense perisomatic input from glutamatergic boutons. In response to a sound, these cells could provide an inhibitory signal to the thalamus that precedes excitation. Here, we demonstrate that colliculothalamic projections arise from four GABAergic cell subtypes. The predominant subtype has a small soma, suggesting slower inhibition that presumably overlaps the arrival of excitatory inputs to the thalamus. The results suggest extensive opportunity for temporal integration of inhibitory and excitatory inputs, and a wider diversity of functions than previously suggested for inhibitory colliculothalamic projections.

## Introduction

Throughout sensory systems, GABAergic cells often make only local projections, defining them as interneurons. GABAergic neurons in the inferior colliculus (IC), a midbrain hub of the auditory system, are unusual because in addition to contacting cells locally they make long-range projections to other nuclei (Winer et al., 1996). GABAergic IC neurons project to the contralateral IC and to the superior colliculus, however the largest GABAergic output pathway targets the medial geniculate body [(MG) the main auditory nucleus of the thalamus; Appell and Behan, 1990; Nakamoto et al., 2013]. Depending on species, 20–40% of IC–MG neurons are GABAergic (Winer et al., 1996; Peruzzi et al., 1997; Mellott et al., 2014a). A previous study describes the inhibitory component of the IC–MG pathway as dominated by large GABAergic (LG) neurons with somas larger than 16.5 µm in diameter and dense axosomatic input from terminals containing vesicular glutamate transporter 2 (VGLUT2; Ito et al., 2009). The other subtype, small GABAergic (SG) cells, have somas smaller than 10.7 µm in diameter, do not receive axosomatic VGLUT2-containing terminals, and are unlikely to project to the MG (Ito et al., 2009). LG cells have been described in a variety of mammalian species and recently in birds, leading to the suggestion that they represent a basic component of auditory pathways across amniotes (Ito and Atoji, 2016). The authors suggest that the large size coupled with dense axosomatic inputs from excitatory terminals allow LG cells to provide short latency inhibition that arrives in the MG before excitation (Peruzzi et al., 1997; Ito and Oliver, 2012). However, many studies find a range of latencies for IC-generated inhibition in the MG, and an IC–MG pathway dominated by inhibition from LG cells is difficult to reconcile with these physiologic data (Hu et al., 1994; Bartlett and Smith, 1999).

Building on LG/SG classification, we described four subtypes of GABAergic neurons in the IC of guinea pigs by combining staining for VGLUT2 perisomatic bouton rings and perineuronal nets (PNs). PNs are aggregates of extracellular matrix molecules implicated in plasticity and high-speed processing (Lander et al., 1997; Corvetti and Rossi, 2005; Blosa et al., 2015). PNs surround subsets of neurons throughout the adult brain, and are often selectively associated with GABAergic cells (Kosaka and Heizmann, 1989; Celio and Blümcke, 1994; Foster et al., 2014). In the IC, PNs surround 44% of GABAergic cells (Foster et al., 2014). Together, the presence or absence of PNs and VGLUT2 rings distinguish four subtypes of GABAergic cells in the IC. “GAD-only” cells, which lack PNs and VGLUT2 rings, have small or medium somas and are the most common subtype of IC GABAergic cell. The second most common are GAD-PN cells, which lack VGLUT2 rings and have medium somas. GAD-PN-VGLUT2 ring cells have medium or large somas, and are surrounded by both markers. Finally, GAD-VGLUT2 ring cells lack PNs, and are the least common (∼2% of GABAergic IC cells; Beebe et al., 2016). We predicted that the largest GABAergic cells associated with VGLUT2 rings and/or PNs were likely to project from the IC to the MG. Further, we hypothesized that our GAD-only cells, which lack PNs and VGLUT2 rings, are unlikely to participate in the IC–MG pathway (and presumably project to other targets or act as local interneurons in the IC).

Here, we tested these hypotheses by making injections of retrograde tracer into the MG of guinea pigs and analyzing retrogradely labeled IC cells for size and GABA subtype. We found that all sizes of GABAergic IC cells project to the MG. Further, all four subtypes of GABAergic cells project to the MG. Finally, the “GAD-only” cells, on average the smallest GABAergic subtype, are the most numerous subtypes in the colliculothalamic pathway. These data suggest that the current model of the colliculothalamic pathway be revised to reflect a multitude of GABAergic subtypes and a temporally diverse pattern of inhibitory inputs to the thalamus.

## Materials and Methods

All procedures were conducted in accordance with the Northeast Ohio Medical University Institutional Animal Care and Use Committee and with NIH guidelines. Results are from nine adult pigmented guinea pigs of either sex from Elm Hill Labs. Animal weights ranged from 407 *g* to 1115 *g*. One of the animals had an injection of Fluoro-Gold retrograde tracer in the left superior colliculus as part of a separate study that yielded little axonal transport and no labeled cells in the IC. Efforts were made to minimize the number of animals and their suffering.

### Surgery

Each animal was anesthetized with isoflurane (4–5% for induction, 1.75–3% for maintenance) in oxygen. The head was shaved and disinfected with betadine (Purdue Products). Atropine sulfate (0.08 mg/kg, i.m.) was given to minimize respiratory secretions, and Ketofen (ketoprofen, 3 mg/kg, i.m.; Henry Schein) was given for postoperative pain management. Moisture Eyes PM ophthalmic ointment (Bausch and Lomb) was applied to each eye to protect the cornea. The animal’s head was positioned in a stereotaxic frame, with the incisor bar set 5.0 mm below interaural zero. Body temperature was maintained with a feedback-controlled heating pad. Sterile instruments and aseptic techniques were used for all surgical procedures. An incision was made in the scalp and the surrounding skin was injected with Marcaine (0.25% bupivacaine with epinephrine 1:200,000; Hospira), a long-lasting local anesthetic. A craniotomy was made over the desired target coordinates using a Foredom High Speed Rotary handpiece (Foredom Electric). Following tracer injection, Gelfoam (Harvard Apparatus) was placed in the craniotomy and the scalp was sutured. The animal was placed in a clean cage, and was monitored until it could walk, eat, and drink without difficulty.

### Retrograde tracers

Fluorescent tracers were deposited in the MG via stereotaxic coordinates. Tracers used were red or green fluorescent Retrobeads [“red beads” (RB) and “green beads” (GB), respectively; undiluted; Luma-Fluor), or fast blue (FB; 5% solution in H_2_O; Sigma-Aldrich, F-5756). The use of several different tracers avoided possible biases associated with any one tracer (e.g., uptake of the tracers can be by different mechanisms). A Hamilton microsyringe (1 or 10 µl; Hamilton) was used to deposit one of the listed tracers into the MG. Each syringe was dedicated to a single tracer. Two to 12 deposits of tracer were made in a given MG (Table 1). The number of deposits and coordinates of each deposit were varied to maximize the number of MG subdivisions included within cases, and within the study overall.

**Table 1.** Summary of tracers, volumes, and stereotaxic coordinates

				Stereotaxic Coordinates		
Case	Tracer	No. of sites	Volume per site, µl	Anterior-posterior	Medial-lateral	Dorsal-ventral
GP731	RB	12	0.1	+5.0 +5.0 +5.3 +5.3	+3.9 +4.4 +3.9 +4.4	+4.0, +4.3, +4.6 +4.0, +4.3, +4.6 +4.0, +4.3, +4.6 +4.0, +4.3, +4.6
GP733	RB	12	0.1	+5.0 +5.0 +5.3 +5.3	+3.9 +4.4 +3.9 +4.4	+4.0, +4.3, +4.6 +4.0, +4.3, +4.6 +4.0, +4.3, +4.6 +4.0, +4.3, +4.6
GP744	GB	8	0.1	+5.0 +5.0 +5.3 +5.3	+3.9 +4.3 +3.9 +4.3	+4.1, +4.6 +4.1, +4.6 +4.1, +4.6 +4.1, +4.6
GP745	CTB	8	0.1	+4.8 +4.8 +5.1 +5.1	+3.9 +4.4 +3.9 +4.4	+4.2, +4.5 +4.2, +4.5 +4.2, +4.5 +4.2, +4.5
GP748	CTB	8	0.1	+4.8 +4.8 +5.1 +5.1	+3.9 +4.4 +3.9 +4.4	+4.2, +4.5 +4.2, +4.5 +4.2, +4.5 +4.2, +4.5
GP749	CTB	8	0.1	+4.8 +4.8 +5.1 +5.1	+3.9 +4.4 +3.9 +4.4	+4.2, +4.5 +4.2, +4.5 +4.2, +4.5 +4.2, +4.5
GP757	FB	8	0.03	+4.5 +4.5 +5.1 +5.1	+3.9 +4.6 +3.9 +4.6	+4.2, +4.8 +4.2, +4.8 +4.2, +4.8 +4.2, +4.8
GP767	FB	2	0.05	+4.8	+4.3	+4.0, +4.4
GP774	FB	2	0.05	+4.8	+4.5	+3.1, +3.6

Tracers were deposited at multiple locations (Number of sites). Each site in an animal received the same volume of tracers (Volume per site). Stereotaxic coordinates (relative to interaural 0) list the locations of each deposit. Each row in the coordinate table corresponds to a single vertical penetration with the microsyringe, located at the given anterior-posterior and medial-lateral coordinates. Tracer was deposited at two or three locations in each penetration, indicated by the dorsal-ventral coordinates in each line. CTB, Cholera toxin B subunit, FB – Fast Blue; GB – green RetroBeads; RB – red RetroBeads.

### Perfusion and tissue processing

After 5–11 d for tracer transport, each animal was deeply anesthetized with isoflurane until breathing stopped and corneal and withdrawal reflexes were absent. The animal was then perfused transcardially with Tyrode’s solution, followed by 250 ml of 4% paraformaldehyde in 0.1 m phosphate buffer, pH 7.4, then 250 ml of the same fixative containing 10% sucrose. The brain was removed and stored in fixative containing 25% sucrose at 4°C overnight. The following day, the cortex and cerebellum were removed and the brainstem was frozen and cut into 40 μm sections in the transverse plane on a sliding microtome. Sections were collected in six series. One series was divided; sections caudal to the superior colliculus were stained for brain nitric oxide synthase (bNOS) to identify IC subdivisions and the remaining rostral sections were stained for cytochrome oxidase (CO) to identify MG subdivisions (Anderson et al., 2007; Coote and Rees, 2008). One or more additional series were stained with a four-color immunofluorescence and histofluorescence procedure to analyze soma size and subtype of GABAergic neurons (Beebe et al., 2016).

For bNOS staining, tissue sections were washed with PBS (0.9% NaCl in 0.01 m phosphate buffer, pH 7.4), then nonspecific staining was blocked by treating sections with a solution of 0.3% Triton X-100 and 10% normal donkey serum in PBS for 1 h at room temperature. Sections were rinsed in PBS, then mounted from a 0.2% gelatin solution onto gelatin-coated slides, allowed to air-dry, and coverslipped with DPX mounting medium (Sigma-Aldrich, 06522).

For CO staining, tissue sections were washed in PBS, then treated with a solution that combined 20 mg of diaminobenzidine hydrochloride (Sigma-Aldrich, D-5906) in 10 ml of dH_2_O with 30 mg of cytochrome c (Sigma-Aldrich, C7752-1G) and 3 *g* of sucrose in 30 ml of 0.1 m phosphate buffer. Sections were incubated in the solution either at 4°C overnight or at 37°C for 3–5 hours. After staining, sections were mounted from a 0.2% gelatin solution onto gelatin-coated slides, allowed to air-dry, and then coverslipped with DPX mounting medium.

For four-color staining, sections were washed in PBS, then permeabilized in a solution containing 0.2% Triton X-100 in PBS for 30 min at room temperature. Nonspecific staining was blocked by treating tissue with 0.1% Triton X-100 and 10% normal goat serum in PBS for 1 h at room temperature. Tissue sections were washed in PBS, then treated with a cocktail of secondary antibodies containing either an AlexaFluor 546-tagged or an AlexaFluor 488-tagged anti-mouse antibody (A10036 and A21202, respectively; to reveal the anti-GAD67 primary), an AlexaFluor 647-tagged anti-guinea pig antibody (A21450; to reveal the anti-VGLUT2 primary), and an AlexaFluor 750-tagged anti-rabbit antibody (A21039; to reveal the anti-NeuN primary; all at 1:100 dilution; Life Technologies) in PBS at room temperature for 1 h. Sections were rinsed in PBS, then mounted from a 0.2% gelatin solution onto gelatin-coated slides, allowed to air-dry, and coverslipped with DPX mounting medium. Antibodies described here have been previously validated in guinea pig IC (Foster et al., 2014; Beebe et al., 2016).

### Experimental design and statistical analysis

Two quadruple-stained transverse sections through a mid-rostro-caudal level of the IC ipsilateral to the MG injection were selected from each case. Each section was outlined using a Neurolucida reconstruction system (MBF Bioscience) attached to a Zeiss AxioImager Z2 microscope (Carl Zeiss MicroImaging). The outline was overlaid onto an adjacent section stained for bNOS, and differential immunoreactivity was used to draw borders between the central nucleus (ICc) and the lateral (IClc) and dorsal (ICd) cortices of the IC (Coote and Rees, 2008). Borders between the layers of the IClc were added using the NeuN stain (Faye-Lund and Osen, 1985). The quadruple-stained section was then remounted in the microscope, illuminated for NeuN, and a “virtual tissue” photomontage of the entire IC was collected at 2 µm depth intervals with a 63× oil-immersion objective (NA = 1.4). The montage was displayed on a Cintiq 21UX interactive pen display (Wacom) attached to the Neurolucida system. The Cintiq stylus was used to manually trace the soma of every NeuN-reactive cell with a visible nucleolus within 4 μm of one cut surface of each section. This depth was chosen as a criterion for analysis because preliminary analysis showed that each of the fluorescent markers penetrated the section at least this far; thus, lack of staining with a given marker is unlikely to be due to inadequate penetration of the staining reagents (Mellott et al., 2014a). The section outline, with its associated NeuN-stained soma outlines, was then aligned to the original section, and each neuron was viewed with the appropriate fluorescence filters to identify expression of the four additional markers (retrograde tracer, a PN, expression of GAD67, or a dense ring of axosomatic VGLUT2-expressing terminals). A soma was considered to have a dense ring of VGLUT2-expressing terminals if >∼75% of the perimeter was covered by VGLUT2-immunopositive puncta. Each neuronal outline was color-coded to indicate which marker(s) it was associated with. A total of 56,236 neurons were outlined and coded, of which 4282 were labeled with retrograde tracer. Neurons with a profile area that overlapped a subdivision outline were excluded from analysis, so as not to be counted twice.

Data including the coded cell type (e.g., GAD67+), perimeter, area, X and Y coordinates of the centroid, and minimum and maximum Feret diameters for each soma outline were exported from Neurolucida into R v3.4.1 for Mac OS X (R Core Team, 2017) for all further analyses. Similarity in proportions of soma sizes and GABA subtypes between the IC overall and the IC–MG pathway were tested with G tests for goodness of fit. Source code for G tests was imported from http://www.psych.ualberta.ca/∼phurd/cruft/g.test.r. The difference in soma profile area between GABA subtypes within the IC–MG pathway was tested with a linear mixed effects model. Case was included as a random factor in models to account for minor variations in soma size that could result from fixation differences. The “lme4” package was used for fitting of linear mixed effects models (Bates et al., 2015), and the “emmeans” package was used for pairwise comparisons (Tukey’s HSD; Lenth, 2018). Soma profile area data were log-normally distributed, so data were log-transformed before statistical testing. Composite plots and box plots were generated in R (for an explanation of the generation of composite plots, see Beebe et al., 2016). Bar graphs were generated in Microsoft Excel.

Photomicrographs were taken using a Zeiss AxioImager Z2 microscope with an attached Apotome II (Zeiss) using Neurolucida software (MBF biosciences). Photographs were taken using structured illumination microscopy with optical sectioning at 0.2 µm depth intervals. Adobe Photoshop (CS6, Adobe Systems) was used to add scale bars, and crop and colorize images. Brightness and contrast levels were adjusted globally when necessary.

## Results

### Retrograde tracer injections included lemniscal and non-lemniscal MG

**Figure 1.**
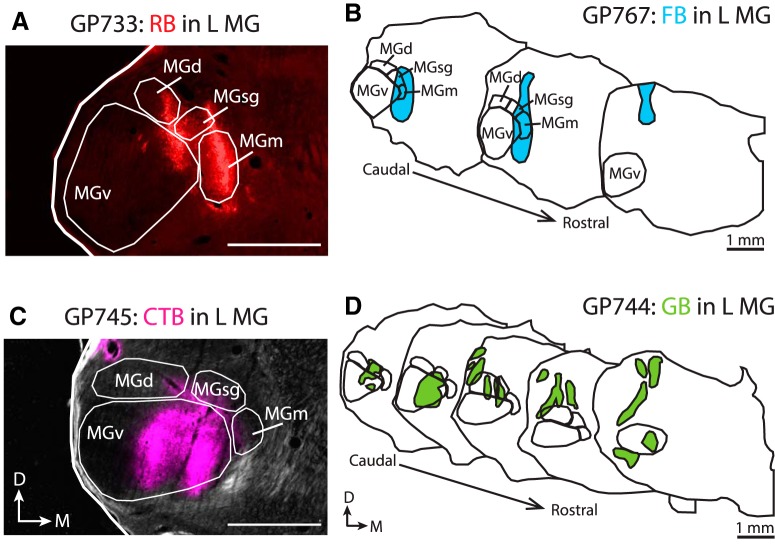
Tracer deposits included lemniscal and non-lemniscal MG subdivisions. Depictions of 4 of the 9 retrograde tracer deposits. ***A***, ***B***, Tracer deposits centered in non-lemniscal regions of the MG. ***C***, ***D***, Tracer deposits largely in lemniscal MGv. ***A***, Photograph showing a single rostrocaudal level of the RB deposit in case GP733. ***B***, Series of tracings showing the rostrocaudal extent of the FB deposit in case GP767. ***C***, Photograph showing a single rostrocaudal level of the CTB deposit in case GP745. ***D***, Series of tracings showing the rostrocaudal extent of the GB deposit in case GP744. Variations like these in tracer deposits ensured that all portions of the IC–MG projection were included in the study. Transverse sections. Scale bars, 1 mm. CTB, Cholera toxin B; D, dorsal; M, medial; MGm, medial subdivision of the MG; MGsg, suprageniculate subdivision of the MG; MGv, ventral subdivision of the MG; RB, red RetroBeads.

Our tracer injections aimed to label all portions of the IC–MG pathway. Figure 1 shows examples of four retrograde tracer injections. In cases GP733 and GP767 (Fig. 1*A*,*B*), the tracer deposits are centered in non-lemniscal MG, while in cases GP745 and GP744 (Fig. 1*C*,*D*), the tracer deposits largely involve ventral MG (MGv), with additional involvement of the dorsal (MGd) and suprageniculate (MGsg) subdivisions in case GP744. Table 2 gives the extent of each injection site, as well as the relative density of retrogradely labeled cells in the IC. As expected from previous studies, there is a correspondence between the MG subdivisions injected and the distributions of the labeled cells in the IC (Mellott et al., 2014b). Briefly, injections into the lemniscal MG (MGv) labeled cells mostly in the ICc, whereas injections in other MG subdivisions gave more widespread labeling, with a majority of cells outside the ICc. Note that each subdivision of the IC was highest in relative density of label in at least one case, confirming that both lemniscal and non-lemniscal IC–MG pathways are well represented in this dataset.

**Table 2.** Extent of injection sites and relative density of retrogradely-labeled neurons

	Extent of injection site				Relative density of retrograde label		
Case	MGv	MGm	MGd	MGsg	ICc	ICd	IClc
GP731	xx	xxx	xxx	x	*	**	***
GP733	xx	xxx	xxx	x	*	***	**
GP744	xxx	—	x	x	***	*	**
GP745	xxx	—	x	x	***	*	**
GP748	—	xxx	x	xx	*	**	***
GP749	—	xxx	—	xx	*	**	***
GP757	xxx	xx	xxx	xxx	**	***	*
GP767	x	xxx	—	xx	***	*	**
GP774	xxx	xxx	xxx	xxx	**	***	*

For each case, the relative involvement of each MG subdivision in the retrograde tracer deposit is shown on the left, and the relative density of retrogradely-labeled cells in the IC is given on the right. For injection site involvement, a dash indicates no involvement, a single “x” indicates minimal involvement, and an increasing number of “x” symbols indicates increasing involvement of the subdivision. For density of retrograde label, all subdivisions of the IC contained retrogradely-labeled cells in each experiment; however, relative density (average cells per mm^2^ across 2 sections) is shown with a single asterisk indicating the lowest relative density of label and three asterisks indicating the highest relative density of retrograde label. MGd: dorsal subdivision of the MG; MGm: medial subdivision of the MG; MGsg: suprageniculate subdivisions of the MG; MGv: ventral subdivisions of the MG; ICc: central nucleus of the IC; ICd: dorsal cortex of the IC; IClc: lateral cortex of the IC.

### A range of sizes of inhibitory cells participates in the IC–MG pathway

Every experiment produced a large number of retrogradely labeled cells in the IC and fewer such cells in numerous subcollicular nuclei, consistent with earlier descriptions in guinea pigs (Schofield et al., 2014a,b). In the IC, the retrogradely labeled cells included both GAD+ and GAD− cells, with the latter forming the majority (Mellott et al., 2014a). The distribution of GAD+ cells across the IC and within individual IC subdivisions is similar to previous reports (Mellott et al., 2014a).

**Figure 2.**
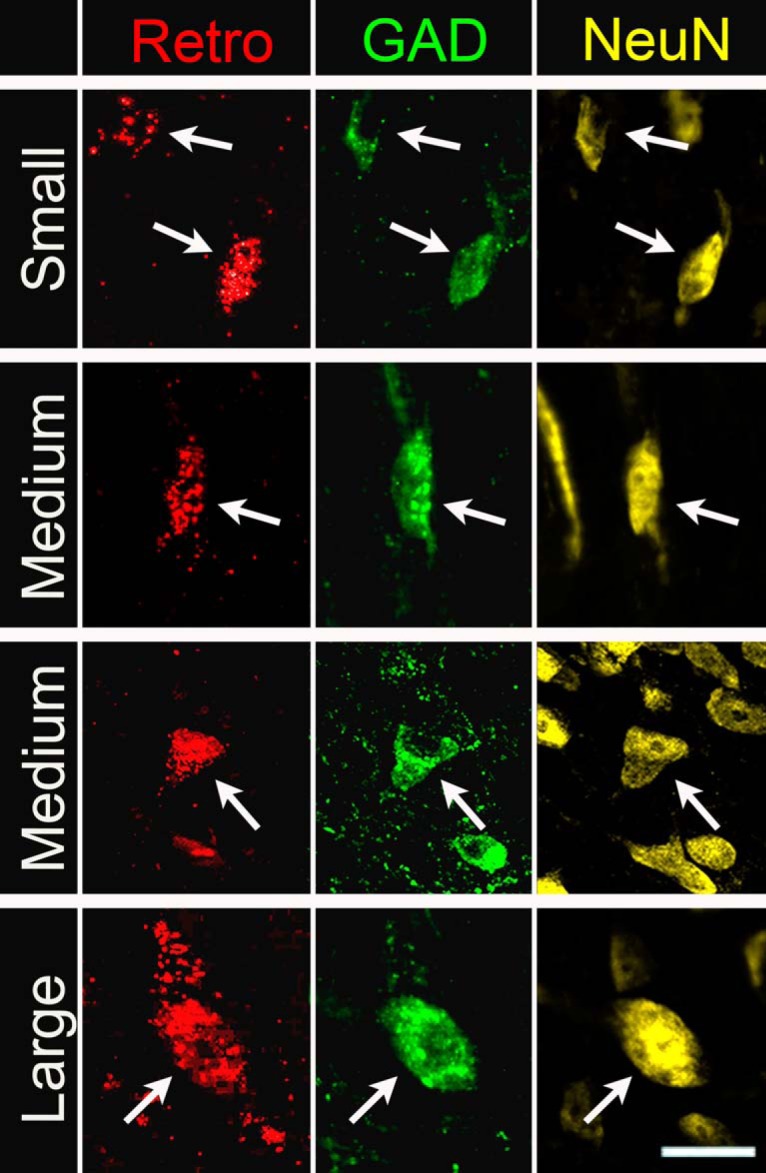
Retrograde tracers label small, medium and large GABAergic IC cells. Photographs of cells (arrows) labeled with retrograde tracer (first column, red, Retro) that were also GAD+ (second column, green) and NeuN+ (third column, yellow). Note that all retrograde tracers are pseudo-colored red for simplicity. The first row shows examples of small GAD+ IC–MG cells (soma profile area <105 µm^2^), the second and third row show examples of medium GAD+ IC–MG cells (soma profile area between 105 and 318 µm^2^), and the bottom row shows an example of a large GAD+ IC–MG cell (soma profile area >318 µm^2^). Scale bar, 20 µm.

**Figure 3.**
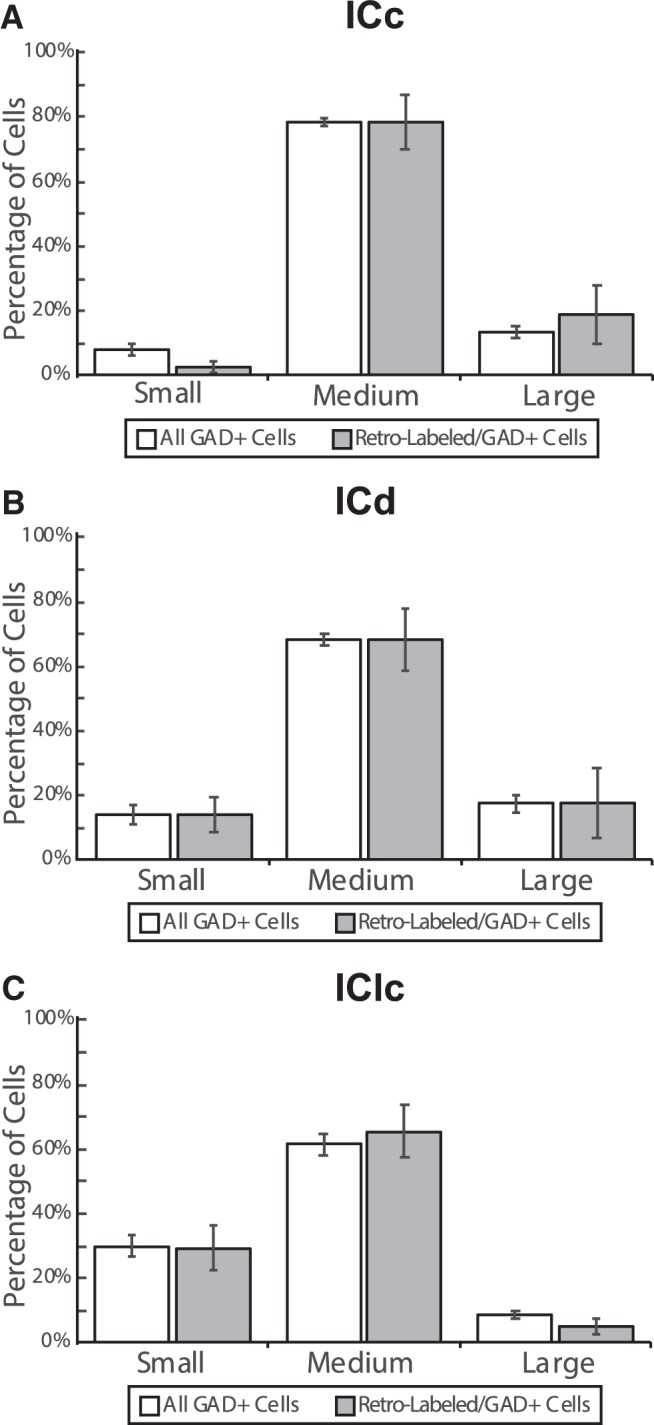
GABAergic retrogradely labeled IC cells exhibit a wide range of soma sizes. Bar graphs showing the proportions of IC GAD+ cells overall (white bars) or the subset that were labeled by retrograde transport from the MG (gray bars). Each population is broken into small, medium, and large soma size categories. The total sample was made up of 6511 GAD+ IC cells, of which 258 were retrogradely labeled. Of these, 3114/100 were in ICc (***A***), 2081/72 were in ICd (***B***), and 1316/86 were in IClc (***C***). Medium cells form the majority of GAD+ cells and GAD+ IC–MG cells in each subdivision. The proportions of GABAergic soma sizes within the IC–MG pathway are reflective of the proportions of GABAergic soma sizes of the overall GABAergic population within the IC. Error bars represent SEM.

In the IC, retrogradely labeled GAD+ cells exhibit a wide range of sizes (Fig. 2). We used criteria established previously (Beebe et al., 2016) to assign GAD+ cells to small, medium, or large categories: small cells have a soma area <105 µm^2^, medium cells have a soma area of 105–318 µm^2^, and large cells have a soma area >318 µm^2^. Cells from all three size categories were labeled by each of the retrograde tracers. Medium-sized cells form the majority of GAD+ cells in each IC subdivision, and are also the most common size among retrogradely labeled GAD+ cells (Fig. 3). Overall, the proportions of soma sizes of GAD+ cells in the IC–MG pathway match the proportions of GAD+ soma sizes present within the IC (*G* test for goodness of fit: *G*_(2)_ = 5.70, *p* = 0.06). This was also true within each of the IC subdivisions individually (ICc: *G*_(2)_ = 1.48, *p* = 0.48; ICd: *G*_(2)_ = 0.64, *p* = 0.73; IClc: *G*_(2)_ = 2.23, *p* = 0.33). We conclude that no category of GAD+ soma size is more or less prevalent in the IC–MG pathway than in the IC overall. Stated another way, in regards to soma size of GAD+ cells, the IC–MG pathway is reflective of the overall GAD+ population within the IC.

### *Four subtypes of inhibitory cells participate in the IC*–*MG pathway*

**Figure 4.**
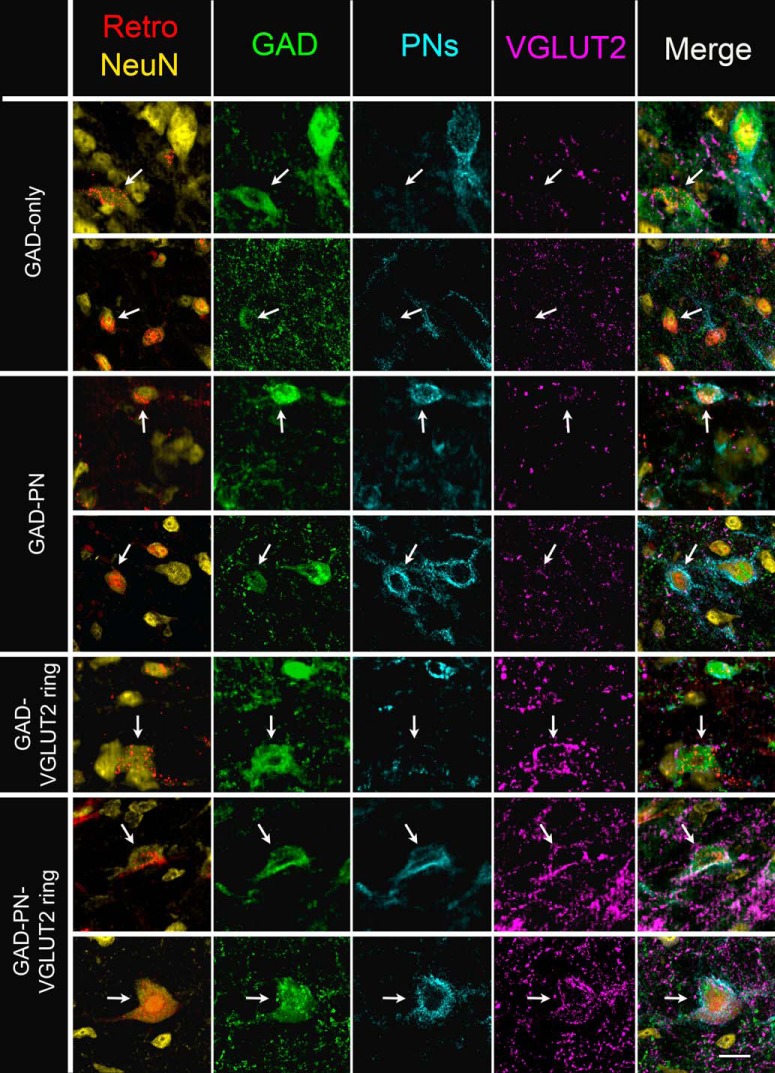
Four subtypes of GABAergic neurons participate in the IC–MG projection. Photographs of neurons (NeuN stain in the first column, yellow) labeled with retrograde tracer (first column, Retro, red) that are also GAD+ (second column, green). In the first two rows (GAD-only), the indicated cells (white arrows) lack both a PN (third column, cyan) and a VGLUT2 ring (fourth column, magenta). In the third and fourth rows (GAD-PN), the indicated cells lack VGLUT2 rings but are surrounded by PNs. In the fifth row (GAD-VGLUT2 ring), the indicated cell lacks a PN but is surrounded by a VGLUT2 ring. In the sixth and seventh rows (GAD-PN-VGLUT2 ring), the indicated cells are surrounded by both PNs and VGLUT2 rings. Note that all tracers have been pseudo-colored red for simplicity. Scale bar, 20 µm.

We previously used extracellular markers (PNs and VGLUT2 rings) to divide IC GAD+ cells into four subtypes (Beebe et al., 2016). These subtypes include “GAD-only” cells (lacking both a PN and a VGLUT2 ring), “GAD-VGLUT2 ring” cells (lacking a PN, but surrounded by a VGLUT2 ring), “GAD-PN” cells (lacking a VGLUT2 ring, but surrounded by a PN), and “GAD-PN-VGLUT2 ring” cells (surrounded by both a PN and a VGLUT2 ring). In addition to the differences in extracellular markers for which they are named, these subtypes also differ in their distribution of soma sizes and their spatial distribution within the IC. Here, we found that all four subtypes of GAD+ cells participate in the IC–MG pathway (Fig. 4).

**Figure 5.**
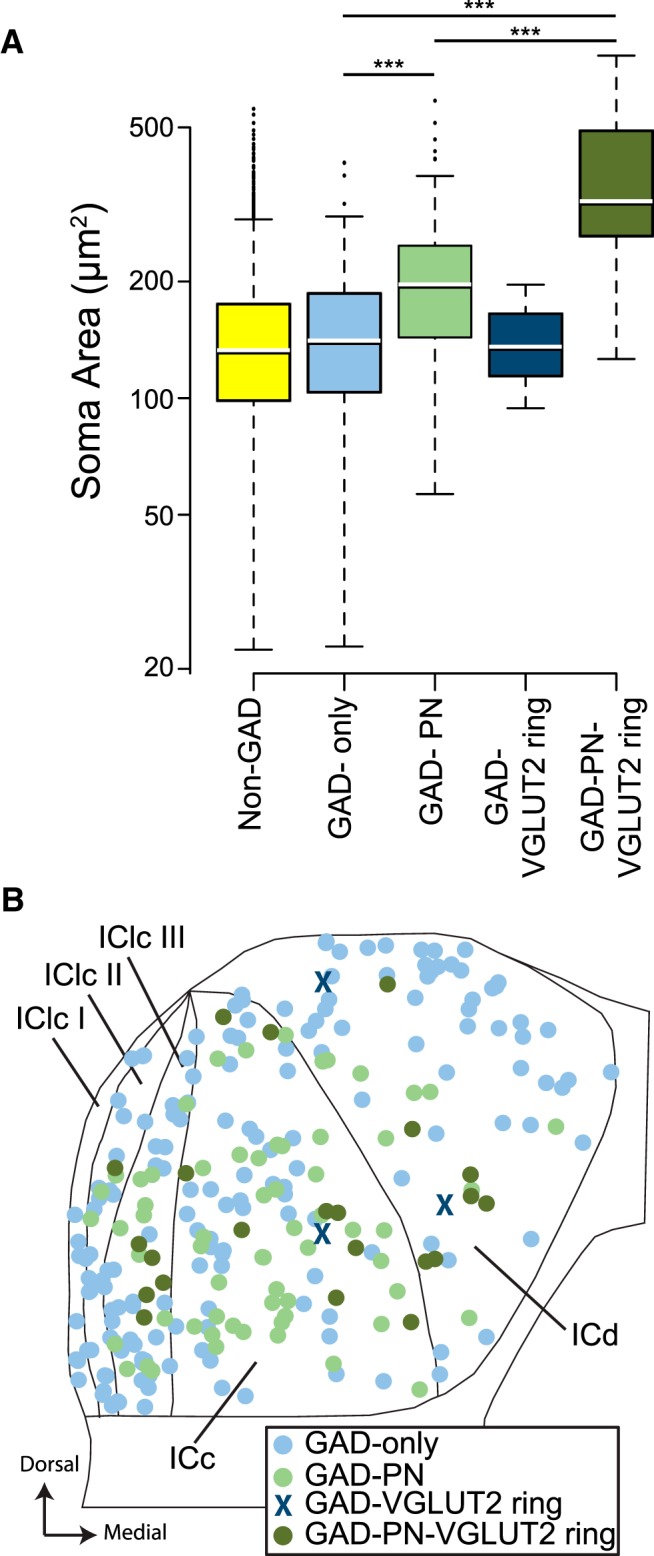
The four subtypes of GABAergic neurons in the IC–MG projection differ in soma size and spatial distribution. ***A***, Box and whisker plot showing the range and median soma profile areas of non-GAD (yellow), GAD-only (light blue), GAD-PN (light green), GAD-VGLUT2 ring (dark blue), and GAD-PN-VGLUT2 ring (dark green) IC–MG cells. On average, GAD-only cell somas are smaller than GAD-PN cell somas, and GAD-PN-VGLUT2 ring cells have the largest somas. Note that the *y*-axis of the box plot is logarithmically scaled. Sample sizes: non-GAD = 4024 cells; GAD-only = 170 cells; GAD-VGLUT2 ring = 3 cells; GAD-PN = 63 cells; GAD-PN-VGLUT2 ring = 22 cells. Three asterisks indicate a significant difference in soma size between two groups at the *p* = 0.001 level. ***B***, Composite plot of all the GAD+ IC–MG projecting cells in this study (cells from two sections each from 6 cases). Light blue markers indicate GAD-only cells, dark blue X’s indicate GAD-VGLUT2 ring cells, light green markers indicate GAD-PN cells, and dark green markers indicate GAD-PN-VGLUT2 ring cells. GAD-only cells are relatively more dense along the lateral and dorsal edges of the IC, whereas GAD+ cells surrounded by PNs and/or VGLUT2 rings are relatively more dense in deeper regions of the IC (ICc and IClc layer III). ICc- central nucleus of the IC; ICd- dorsal cortex of the IC, IClc I, II, and III, Layers I, II, and III of the lateral cortex of the IC.

The differences in soma size and in distribution among IC subdivisions previously reported between the four GABAergic subtypes are also present within the IC–MG pathway. As shown in Figure 5*A*, MG-projecting GAD-PN-VGLUT2 ring somas are larger on average than MG-projecting GAD-PN somas (Fig. 5*A*, compare dark green to light green; mean profile areas of 378 and 213 µm^2^, respectively), and both are larger than MG-projecting GAD-only somas (Fig. 5*A*, light blue; mean profile area of 148 µm^2^). Only three GAD-VGLUT2 ring neurons were present in our sample of 258 GAD+ IC–MG neurons (Fig. 5*A*, dark blue), making comparisons involving this group difficult. However, for the other three groups, a linear mixed effects model of the data were constructed, and a likelihood ratio test showed that there was a significant relationship between GAD subtype and soma profile area (χ^2^_(2)_ = 71.92, *p* = 2.42 × 10^−16^). Pairwise comparison using a Tukey’s HSD showed significant differences in soma profile area between the GAD-only and GAD-PN-VGLUT2 ring groups (*p* < 0.0001), between the GAD-only and the GAD-PN groups (*p* < 0.0001), and between the GAD-PN and GAD-PN-VGLUT2 ring groups (*p* = 0.0001). As shown in Figure 5*B*, the four subtypes of MG-projecting GAD+ cells also differ in their spatial distribution: GAD-only cells (light blue symbols) are found in all areas and are the dominant subtype around the edges of the IC in the ICd and IClc, whereas GAD+ cells surrounded by PNs (green symbols) or VGLUT2 rings (X and dark green symbols) are more numerous in the ICc and in the deeper parts of the IClc and ICd (i.e., adjacent to the ICc).

**Figure 6.**
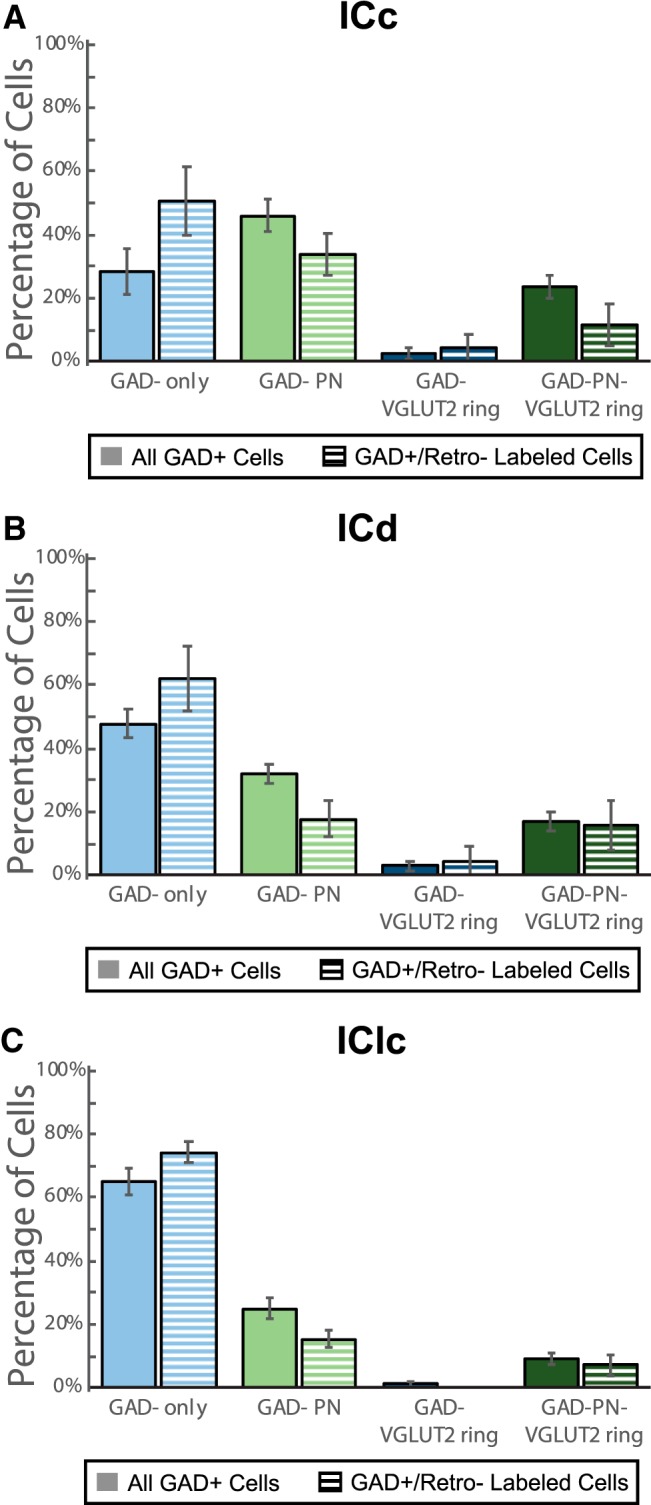
Retrogradely-labeled IC cells comprise four GABAergic subtypes. Bar graphs showing the proportions of IC GAD+ cells overall (solid bars) or the subset that were labeled by retrograde transport from the MG (striped bars). The cells were classified as GAD-only (light blue), GAD-PN (light green), GAD-VGLUT2 ring (dark blue), or GAD-PN-VGLUT2 ring (dark green) in each of the three main IC subdivisions. The total sample was made up of 6511 GAD+ IC cells, of which 258 were retrogradely labeled. Of these, 3114/100 were in ICc (***A***), 2081/72 were in ICd (***B***), and 1316/86 were in IClc (***C***). In each IC subdivision, the proportions of GAD subtypes within the IC–MG pathway were significantly different from the proportions of GAD subtypes within the IC overall, with GAD-Only cells overrepresented in the IC–MG pathway. Error bars represent SEM.

Having determined that all four subtypes of GAD+ cells project to the MG, we were interested in whether the proportion of each subtype in the IC–MG pathway simply reflects the abundance of that subtype in the IC. At one extreme, if every IC GABAergic cell projects to the MG then the populations, and thus the proportions, are identical. However, as described in the Introduction, previous studies concluded that GABAergic cells in the IC–MG pathway are primarily LG cells (with large somas and a VGLUT2 ring; Ito and Oliver, 2012). GAD-only cells, with a small or medium soma and no VGLUT2 ring or PN, are a substantial proportion of the GABAergic population in the IC but would be expected to contribute little to the IC–MG pathway. To examine this issue, we compared the proportions of GABAergic cell subtypes in the IC–MG pathway with the overall GAD+ population in the IC. Because sample sizes of the two VGLUT2-ringed groups were small, the GAD-VGLUT2 ring and GAD-PN-VGLUT2 ring groups were pooled for statistical analysis. A *G* test for goodness of fit showed a significant difference between the proportions of the GABAergic subtypes present within the IC–MG pathway versus the proportions in the IC overall (*G*_(2)_ = 52.24, *p* = 4.54 × 10^−12^). Differences were also apparent in each of the IC subdivisions individually (ICc: *G*_(2)_ = 29.88, *p* = 3.25 × 10^−7^; ICd: G_(2)_ = 10.30, *p* = 0.006; IClc: *G*_(2)_ = 6.09, *p* = 0.05). In the ICc, GAD-PN cells are the most common GABAergic cell subtype (Fig. 6*A*, solid light green bar), but GAD-only cells were the most common GABAergic subtype in the IC–MG pathway (striped light blue bar). In both the ICd and the IClc, GAD-only cells are the most common GABAergic subtype (Fig. 6*B*,*C*, solid light blue bars), and this subtype formed an even higher proportion of the IC–MG pathway from these subdivisions. We conclude that, in regards to subtypes of GABAergic cells, the IC–MG pathway is not completely reflective of the overall GABAergic population within the IC; rather, GAD-only cells are overrepresented and GAD+ cells surrounded by PNs and VGLUT2 rings are underrepresented in the IC–MG projections from all three IC subdivisions.

## Discussion

**Figure 7.**
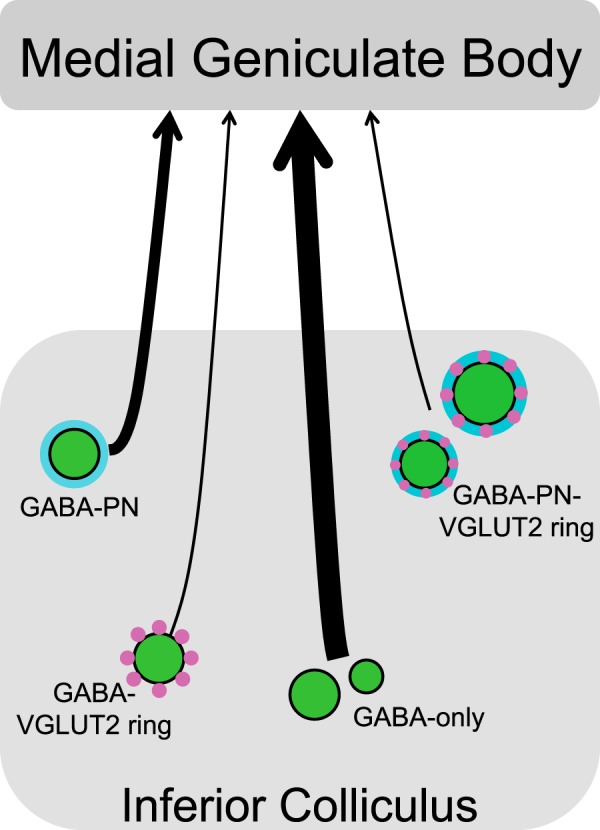
A range of soma sizes and subtypes of cells makes up the GABAergic portion of the IC–MG projection. Black arrows indicate the GABAergic IC–MG projection, with the weight of each arrow reflecting the proportion of the pathway that is made up of each GABAergic subtype. Green circles represent small, medium, and large GABAergic IC cells; subtypes are distinguished by the presence of perineuronal nets (cyan) and/or perisomatic rings of VGLUT2 immunopositive boutons (magenta).

We found that the inhibitory projections from the IC to the MG originate from four different subtypes of GABAergic IC cells that exhibit a wide range of soma sizes (Fig. 7). These results expand current views of the IC–MG inhibitory pathway in several ways. First, soma sizes of GABAergic cells cover the full range of GABAergic soma sizes present in the IC. Moreover, the proportions of GABAergic cell sizes in the IC–MG pathway mirror the proportions of GABAergic soma sizes in the IC overall. This observation contradicts the view that large GABAergic IC cells are the sole source of GABAergic projections to the MG. Second, four subtypes of GABAergic cells project in the IC–MG pathway, challenging the view that the GABAergic projection is dominated by a single subtype. Finally, the most numerous cell type in the IC–MG pathway is a type that has neither a PN nor a perisomatic ring of VGLUT2+ boutons. GABA cells surrounded by a VGLUT2 ring, with or without a PN, were observed in the present study, but constitute a minority in the pathway. The presence of multiple subtypes of GABAergic neurons within the IC–MG pathway is consistent with multiple functions for inhibitory projections from the IC to the MG (discussed below). Although physiologic properties of the large GABAergic cells have been investigated (Geis and Borst, 2013), the present findings show a predominance of GABAergic cells with small or medium size somas that may play a more substantial role than the large cells in the IC–MG pathway. The small and medium GABAergic cells have yet to be characterized physiologically.

### Technical considerations

A major difference with previous studies was our observation of many small and medium GABAergic tectothalamic cells. Could the discrepancy be due to technical factors? The current study used staining for NeuN to identify neurons. The specific criterion of a visible nucleolus ensured that a given profile was a cross-section through the central part of a neuron rather than a tangential shaving through a larger cell. If nucleoli were not visible in the staining techniques used in previous studies, smaller cells could have been dismissed as shavings of larger cells, leading to an overestimation of larger cells. Another main difference is that the current study used several different types of retrograde tracers. Different retrograde tracers diffuse differently at injection sites and can have different labeling efficiencies (Schofield et al., 2007). Each of the tracers used here labeled GABAergic cells of all size categories, supporting the generality of our conclusions.

### *Large GABAergic cells contribute to the IC*–*MG pathway across species and likely underlie fast inhibition of MG cells*

The current model of the IC–MG pathway emphasizes a role for the largest GABAergic cells in the IC (Ito and Oliver, 2012). Our data demonstrate that such cells contribute to the IC–MG pathway in guinea pigs, as they do in several species of mammals and birds (Ito and Atoji, 2016). These large cells likely provide for fast inhibition of their thalamic targets in the MG. Geis and Borst (2013) studied GABAergic cells *in vivo*, using two-photon microscopy in mice. They concluded that large GABAergic cells form a distinct physiologic subtype in the IC. The present study confirms the presence of large GABAergic cells with dense perisomatic VGLUT2 inputs (i.e., rings) in the tectothalamic pathway in guinea pigs. Such large cells likely underlie the early inhibition of MG cells following electrical stimulation of the brachium of the IC (Bartlett and Smith, 1999). Geis and Borst (2013) demonstrated rapid acoustic activation of the large GABAergic cells, supporting the idea of fast inhibition to the MG in response to acoustic stimulation. The authors also concluded that large GABAergic cells constitute a small minority of the IC GABAergic population.

### *Small and medium GABAergic cells dominate the IC*–*MG pathway*

In the present study, we show that small and medium GABAergic cells outnumber the large GABAergic cells. Dominance of small and medium GABAergic cells in the IC–MG pathway is consistent with previous studies. Winer et al. (1996) showed that GABAergic IC–MG cells constitute a wide range of sizes (and probably multiple dendritic morphologies) in cats. Saint Marie et al. (1997) emphasized the presence of large GABAergic axons in the brachium of the IC in cat, but in fact, the majority (>75%) of GABAergic axons in the brachium were under 2 µm in diameter. Small and medium GABAergic IC cells also project to the MG in rats (Ito et al., 2009), but these cells have not been the focus of recent studies.

The small and medium cells are presumably associated with smaller axons and slower conduction of inhibition to the MG than that associated with the large cells. This is consistent with morphologic data showing many small tectothalamic axons in the MG (Bartlett et al., 2000) and with *in vitro* stimulation of tectothalamic axons in the brachium of the IC, which leads to inhibition that can arrive at MG cells before or after excitatory inputs (Bartlett and Smith, 1999; Venkataraman and Bartlett, 2013). The *in vitro* studies also demonstrate multiple forms of integration, with different amounts of excitation and inhibition and varied temporal patterns of their arrival in MG cells. The present results raise the question of whether different MG cells get inputs from different subtypes of GABAergic cells. Glutamatergic IC cells, the source of tectothalamic excitation, appear to comprise at least two subtypes (Bartlett and Smith, 1999; Ito and Oliver, 2012), so there are multiple combinations by which MG cells could integrate the different subtypes of excitatory and inhibitory inputs. The complexity implied by the combinatorial options may reflect the wide range of auditory responses associated with MG cells in general, and with inhibition in the MG in particular (Aitkin and Dunlop, 1969; Allon et al., 1981; Hu et al., 1994; Bartlett and Smith, 1999; Antunes et al., 2010; Anderson and Linden, 2011).

### Distinctions between the GABAergic subtypes

The identification of functionally relevant subtypes is critical, but the functions are not always easily related to useful markers. In neocortex, GABAergic subtypes can be divided into three major subgroups that differ in a long list of attributes. Among these attributes, the expression of parvalbumin, serotonin receptor type 3A, and vasoactive intestinal polypeptide serve as selective markers for the three groups (Markram et al., 2004). The clarification of subtypes has yet to reach a unified state for IC GABAergic cells. Nonetheless, some of the markers carry implications for GABAergic functions. As discussed above, VGLUT2 rings in the IC have been associated with fast activation by acoustic stimuli and fast transmission of inhibition to the thalamus. PNs provide other possible insights into subtype differences (see Discussion in Beebe et al., 2016). For example, PNs have been associated with regulation of neuronal excitability and with inhibition of structural plasticity and possible support of synaptic plasticity (Dityatev et al., 2007; Vigetti et al., 2008; see Discussions in Karetko and Skangiel-Kramska, 2009; Sonntag et al., 2015; Beebe et al., 2016). GABAergic subtypes with or without PNs could be expected to show different degrees of plasticity, perhaps related to signal salience or top-down modulation of the auditory brainstem, or effects of neuromodulatory inputs (Schofield and Hurley, 2018). Sottile et al. (2017a,b) demonstrated nicotinic receptors associated with GABAergic tectothalamic projections, and showed further that these receptors can change during aging. The authors commented on nicotinic receptor subunits expressed in the large GABAergic IC cells, but also showed evidence for such expression in smaller GABAergic cells. Combining these observations with the present results suggests that cholinergic modulation may be affecting multiple subtypes of GABAergic cells and thus multiple components of the IC–MG pathway. Further insights into cholinergic modulation (and GABAergic function in general) during aging may benefit from identification of GABAergic cell subtypes. PNs have also been associated with a resistance to oxidative stress (Cabungcal et al., 2013), raising the possibility that different GABAergic subtypes respond differently during stress; oxidative stress could conceivably result from tissue damage or from changes in input associated with altered cochlear function (such as in aging or acoustic trauma).

A key step in future studies of IC GABAergic subtypes will be to correlate the different types of criteria, such as physiologic characteristics and molecular markers (see discussion in Schofield and Beebe, 2018). Differences in local axons may also prove important. In neocortex, different axon morphologies of GABAergic subtypes underlie trans-columnar versus intracolumnar inhibition (Bacci et al., 2005). This allows differential modulation of GABAergic subtypes to alter information flow through the cortical layers. Differences in the axonal projections of GABAergic subtypes in the IC could also allow for modulatory control of information flow within the IC. We hypothesize that the GABAergic subtypes in the IC have different patterns of local axon arborizations, but data on this issue are currently unavailable. Ultimately, the goal will be to identify a unifying set of characteristics that reflect different functional roles, such as has been accomplished for GABAergic interneurons in neocortex and hippocampus (Lawrence, 2008; Rudy et al., 2011).

### Conclusions

Recent studies of GABAergic cells in the IC–MG pathway have focused on a single subtype of cell characterized by a large soma and dense glutamatergic inputs, implying a monolithic role of fast inhibition to the MG. The present results indicate, first, that the majority of GABAergic IC–MG cells have small or medium somas. This implies that the arrival of ascending inhibitory inputs to the thalamus likely overlap the arrival of ascending excitatory inputs, offering opportunities for multiple forms of integration. In addition, the GABAergic IC–MG cells include members of all four subtypes of GABAergic cell in the IC, supporting the idea that the inhibitory projections serve multiple functions. The GABAergic subtypes differ in their glutamatergic inputs and presence or absence of a PN, suggesting different roles for the subtypes in normal auditory processing and possibly different roles during aging or in response to damage.

## Synthesis

Reviewing Editor: Leonard Maler, University of Ottawa Decisions are customarily a result of the Reviewing Editor and the peer reviewers coming together and discussing their recommendations until a consensus is reached. When revisions are invited, a fact-based synthesis statement explaining their decision and outlining what is needed to prepare a revision will be listed below. The following reviewer(s) agreed to reveal their identity: NONE. Note: If this manuscript was transferred from JNeurosci and a decision was made to accept the manuscript without peer review, a brief statement to this effect will instead be what is listed below.

The initial reviews were both very positive. The only criticism of this Ms is that it was 'not of general interest'.

The authors have now discussed the relevance of their neuroanatomical data to the function of the inhibitory component of the IC to MGB projection.

I carefully read this section and believe that it should serve as a good starting point for neurophysiologists studying the ascending auditory pathway.

I consider this revised Ms to be suitable for publication in eNeuro as it stands.

But, I also have a suggestion for the authors.

Is a GABAergic component of brainstem to thalamus sense input unique to the auditory system or is it found for other senses.

That is: (a) are there brainstem somatosensory GABAergic neurons that project to the thalamic somatosensory nuclei?

And, equivalently, are there GABAergic superior colliculus neurons that project to pulvinar?

I really don't know the answers to either question. The authors very elegant study made me wonder: is the GABA ascending input a general feature of sensory thalamic input or a special feature of the auditory system.

I think adding just 2 or 3 lines raising this question and either answering it or stating that this has not been investigated would make this Ms of more general interest

What would be (for me) a real a 'clue to function' would be if this was an exclusive feature of the auditory system.

I again emphasize that I do not require this addition; this is merely a suggestion for making this Ms more relevant to a wider range of neuroscientists - those interested in thalamic sensory processing.
